# Hospital workers mental health during the COVID-19 pandemic: methods of data collection and characteristics of study sample in a university hospital in Milan (Italy)

**DOI:** 10.1186/s12874-021-01355-1

**Published:** 2021-08-10

**Authors:** A Fattori, F Cantù, A Comotti, V Tombola, E Colombo, C Nava, L Bordini, L Riboldi, M Bonzini, P Brambilla

**Affiliations:** 1 Occupational Health Unit, Foundation IRCCS Ca’ Granda Ospedale Maggiore Policlinico, Milan, Italy; 2 Department of Pathophysiology and Transplantation, University of Milan, Milan, Italy; 3 Department of Neurosciences and Mental Health, Foundation IRCCS Ca’ Granda Ospedale Maggiore Policlinico, Milan, Italy; 4 Department of Clinical Science and Community Health, University of Milan, Milan, Italy

**Keywords:** Workers’ health surveillance, Occupational stress, COVID-19 psychological impact, Post-traumatic stress disorder, COVID-19 research methods, Risk assessment, Longitudinal studies

## Abstract

**Background:**

The COVID-19 pandemic is currently a severe challenge for healthcare workers, with a considerable impact on their mental health. In order to focus preventive and rehabilitation measures it’s fundamental to identify risk factors of such psychological impairment. We designed an observational longitudinal study to systematically examine the psychological wellbeing of all employees in a large University Hospital in Italy, using validated psychometric scales in the context of the occupational physician’s health surveillance, in collaboration with Psychiatric Unit.

**Methods:**

The study started after ethical approval in August 2020. For each worker, the psychological wellbeing is screened in two steps. The first level questionnaire collects sociodemographic characteristics, personal and occupational COVID-19 exposure, worries and concerns about COVID-19, general psychological discomfort (GHQ-12), post-traumatic stress symptoms (IES-R) and anxiety (GAD-7). Workers who score above the cut-off in at least one scale are further investigated by the second level questionnaire composed by PHQ-9, DES-II and SCL-90. If second level shows psychological impairments, we offer individual specialist treatment (third level). We plan to follow-up all subjects to monitor symptoms and possible chronicization; we aim to investigate potential risk factors through univariate analysis and multivariate logistic regressions.

**Results:**

Preliminary results refer to a sample of 550 workers who completed the multi-step evaluation from August to December 2020, before vaccination campaign started. The participation rate was 90%. At first level screening, 39% of the subjects expressed general psychological discomfort (GHQ-12), 22% post-traumatic stress symptoms (IES-R), and 21% symptoms of anxiety (GAD-7). Women, nurses, younger workers, subjects with COVID-19 working exposure and with an infected family member showed significantly higher psychological impairment compared to colleagues. After the second level screening, 12% and 7% of all workers showed, respectively, depressive and dissociative symptoms; scorings were significantly associated with gender and occupational role. We are currently extending sample size and evaluating subjects over a period of further 12 months.

**Conclusions:**

The possibility to perform a systematic follow-up of psychological wellbeing of all hospital workers, directly or indirectly exposed to pandemic consequences, constitutes a unique condition to detect individual, occupational, and non-occupational risk factors for psychological impairment in situations of prolonged stress, as well as variables associated with symptoms chronicization.

## Background

The pandemic of coronavirus disease 19 (COVID-19), caused by the severe acute respiratory syndrome coronavirus 2 (SARS-CoV-2), represents a severe and not previously experienced challenge for healthcare systems. Particularly, Northern Italy has been the epicenter of the first outbreak of COVID-19 in a Western country.

The hospital in which we developed our study, the Foundation IRCCS Ca’ Granda Ospedale Maggiore Policlinico in Milan, Italy, was completely reorganized to face properly both the rise of new cases and the large number of patients with the same disease accessing to intensive treatments; this led to a massive yet efficient reorganization of the entire healthcare system [1]. Healthcare workers, for being exposed to a rapid and intense stress and for the consequent fear for their health, have lived an experience which was compared to a war event in terms of psychological impact [2]. Technical-administrative staff was also involved in a complex reorganization of the whole hospital structure, facilities and procedures.

These events have been associated to a deterioration of psychological health and have been recognized as potential triggers for various mental-health diseases, including first episodes of psychosis [3] and post-traumatic stress disorder (PTSD) [4, 5]. Psychological impairment has particularly severe consequences on healthcare workers also impacting public health by diminishing psychological resources, working abilities and competences, potentially causing inappropriate, negligent and careless behaviors [6].

PTSD can be easily treated if diagnosed in its early phases. A rapid diagnosis is fundamental, but it requires an active surveillance of workers from specialized experts, in order to recognize first and under-threshold symptoms, prevent the most severe forms and thus treat affected workers [7, 8].

Moreover, occupational stress, long-working hours and various concerns regarding personal and familiar safety could trigger or worsen anxious and/or depressive disorders, which must be properly recognized, treated and rehabilitated.

Methods

### Study aims

We set a multi-step evaluation of mental health in all workers of our hospital, in order to:

- 1. evaluate with standardized tests the psychological wellbeing with a structured medical-assisted interview in the context of occupational health surveillance;
- 2. propose, when first-level tests showed indicators of psychological impairment, a second-level questionnaire to better assess possible psychological distress;
- 3. offer a specialist evaluation (i.e. third-level step) to whom showed specific symptoms at the second-level questionnaire, followed, if needed, by an individual psychological support and/or psychiatric treatment;
- 4. follow up workers over an extended time period, with a revaluation after 6 months for workers with sub-optimal psychological wellbeing at first level, and after 12 months for others, in order to evaluate trends in psychological burden, recognize delayed onset of symptoms and evaluate the efficacy of specialist treatments.

### Study design and population

The study design is observational and longitudinal, non-pharmacological.

Our population of interest is composed by all workers in Foundation IRCCS Ca’ Granda Ospedale Maggiore Policlinico, as every person has been differently involved in the COVID-19 pandemic, with a significant a priori impact of the event, as described in our rationale.

From July 2020 onwards all workers have been invited to participate, independently from age, sex, department and job title. The only two exclusion criteria were being employed after the beginning of the study and the refusal to sign the informed consent; there were no exclusion criteria on pre-existing pathologies, aiming to include the overall and most general pool of population.

The study is conducted jointly by the units of Occupational Medicine and Psychiatry. Our first-level evaluation is organized within the medical surveillance required by Italian Legislation in terms of occupational safety (i.e. Legislative Decree n.81/2008) and extended to workers without an already scheduled occupational physician visit. We are planning to evaluate approximately 3.000 workers in a period of 24 months covering the majority of the current working force of our Hospital.

The participation is voluntary, and an extended informed-consent form is signed before the first-level evaluation. Formal ethical approval has been obtained by the Hospital ethical committee in July 2020. The hospital covered the full cost of the study.

### Collected data and questionnaires

**Fig. 1:**
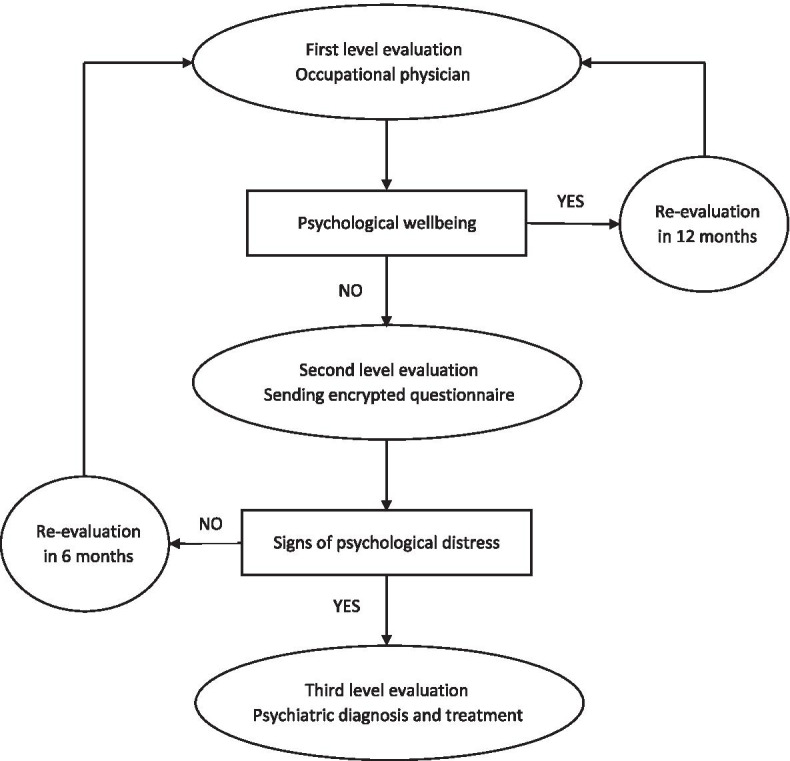
Study Flowchart

The study steps are graphically summarized in Fig. 1.

1. First level questionnaire is collected directly on digital support and consists of three sections. The first section includes socio-demographic questions (age, sex), occupational data (occupational role, hospital unit/department, job seniority regarding current role/current hospital/healthcare system; working experience in COVID-19 areas, with specific details on intensity and time spent) and clinical questions regarding chronic conditions and habitual medications, with a distinction for those taken after the onset of the pandemic (i.e. March, 2020).

The second section contains brief self-administered screening tools aimed at preliminarily assessing potential impairments of worker’s psychological heath:

- The General Health Questionnaire (GHQ-12) [9]. GHQ-12 is a widely used scale for assessing psychological distress and short-term changes in mental health; due to its massive use in in clinical practice, epidemiological and psychological research, it allows comparisons with normative data and findings from different population settings. We adopted the dichotomous scoring method (0–0-1–1) as suggested by the authors [10]; as the cut-off points we chose above or equal to 4, a common measure in literature, which offers a balance between sensitivity (i.e. detecting a correct proportion of people who have a psychiatric disorder and who score above a cut-off) and specificity (i.e. the proportion of people without a psychiatric disorder who score below a cut-off on the same instrument) while limiting the over-diagnosis of patients who are not likely to have a disorder (i.e. false positives) [11, 12]. For this study we adopted the validated Italian version [13, 14].

- Impact of Event Scale-Revised (IES-r). IES-r is one of the most common used questionnaire for assessing post-traumatic stress symptoms across different cultures, settings and types of trauma [15]. A brief description guides subjects to answer the following questions by assessing their subjective responses related to COVID-19 emergency in the previous 7 days; IES-r has 22 questions exploring intrusion (intrusive thoughts, nightmares, intrusive feelings and imagery, dissociative-like re-experiencing), avoidance (numbing of responsiveness, avoidance of feelings, situations, and ideas), and hyperarousal symptoms (anger, irritability, hypervigilance, difficulty concentrating, heightened startle) on a 5 points Likert scale ranging from 0 (not at all) to 4 (extremely). As IES-r is not a proper diagnostic tool for PTSD, higher scores are representative of greater distress and may indicate the need for further evaluation. However, a total score of 33 on the IES-r yielded diagnostic sensitivity of 0.91 and specificity of 0.82 [16]. The Italian version has also shown optimal psychometric properties and validity [17].

- Psychosocial Safety Climate (PSC) [18]. PSC refers to employees’ shared perceptions regarding policies, procedures and practices for the protection of psychological health and safety developed by their organization management. On a 5-point Likert scale ranging from 1 (strongly disagree) to 5 (strongly agree), PSC investigates perceptions on four domains: (1) management support and commitment for stress prevention; (2) management priority to psychological health and safety versus productivity goals; (3) organizational communication in relation to psychosocial risks; and (4) employees participation and involvement in stress prevention. PSC is associated to psychological distress via job demands and predicts engagement through its positive relationship with resources. Moreover, evidence suggests associations with emotional exhaustion among healthcare workers and a link with patients safety in healthcare settings [19, 20]. For this study we adopted a recent 4 item-version of PSC scale which has shown optimal psychometrics and predictive validity [21].

- Generalized Anxiety Disorders (GAD-7) [22]. GAD-7 is a valid and commonly used screening tool for assessing anxiety symptoms and disorders in both research and clinical practice. It consists of seven items assessing frequency of different concerns and worries experienced during the previous two weeks. With robust psychometric properties and strong validity, a score of 10 or greater represents a reasonable cut-off point to identify cases of GAD; increasing scores on the GAD-7 are also strongly associated with multiple domains of functional impairment and disability.

The third sections includes questions about exposure to COVID-19 and consequent health concerns/ believes: being positive to COVID-19 and duration of the condition, being in quarantine and duration, having colleagues/family members positive to the COVID-19, having family members hospitalized or deceased because of the COVID-19, personal concern of infecting family members, experience of social discrimination outside the hospital, changes in family’s habits, thoughts about changing job, fear for their own safety, experience of moral injury at work, previous experience with infectious diseases, previous training about Personal Protective Equipment (PPE), utilizations of psychological support given by the hospital.

Results of all psychometric scales are immediately available to the occupational physician who, if any test is above the abovementioned cut-off, informs the subject and offers the second-level assessment.

2. The second-level questionnaire is completed by subjects remotely and with an individual link sent by email to each worker, with a personal password given by the physician on the occasion of the first-level evaluation, in order to obtain complete confidentiality of collected data and personal information. The completed and encrypted questionnaires (without individual personal data to ensure confidentiality) are then sent to the Unit of Psychiatry for interpretation and to state if the third-level evaluation is needed.

The second level questionnaire contains specific scales to further investigate psychopathological symptoms and disorders:

- Symptom Checklist-90-Revised (SCL-90-R) [23] is a self-administered scale for the evaluation of psychiatric symptomatology. The checklist consists of nine primary symptom dimensions, including: somatization (SOM), obsessive–compulsive (O-C), interpersonal sensitivity (INT), depression (DEP), anxiety (ANX), hostility (HOS), phobic anxiety (PHOB), paranoid ideation (PAR) and psychoticism (PSY). It includes three global indices of psychological distress: Global Severity Index (number of symptoms endorsed and intensity of distress), Positive Symptom Distress Index (average level of distress for those items that were endorsed; exaggerating or attenuating response style), and Positive Symptoms Total (total symptoms endorsed/breadth of distress). SCL90 has 90 items with a five-point scale from 0 (Not at All) to 4 (Extremely) specifying how much each symptoms has bothered them during the past 7 days. It provides a subjective report of current distress and can be administered repeatedly to track changes in symptoms over time. Each of the dimensions has a relative score calculated as an average of the answered questions, and in general are considered of interest average scores equal to or greater than 1; a global index is also calculated (GSI Global Score Index) as an average score of all questions answered in the test. For this study we use the Italian version [24].

- The Dissociative Experiences Scale II (DES II) [25, 26]. Dissociative symptoms are frequently found in the aftermath of trauma and occurs to some degree in individuals without mental disorders and is thought to be more prevalent in persons with major mental illnesses. The DES II has been developed to offer a means of reliably measuring dissociation in normal and clinical populations. It consists of 28 items that describe common dissociative experiences. Subjects are asked to sign the frequency (from 0 to 100%) with whom a specific dissociative symptom was ever experienced; scores of 20 or more are consistent with post-traumatic or dissociative disorders. DES-II has good psychometric properties, excellent internal consistency, good test–retest reliability, and good convergent validity [27], which are all preserved in the Italian version [28].

- Patient Health Questionnaire-9 (PHQ-9) [29]; PHQ-9 is a self-administered version of the Primary Care Evaluation of Mental Disorders (PRIME-MD) [30], a screening instrument designed for primary care using DSM-IV criteria [31]. The PHQ-9 is aimed at assessing depression disorder by scoring each of the 9 DSM-IV criteria from ``0'' (not at all) to ``3'' (nearly every day) and is also a reliable and valid measure of depression severity. Given these characteristics plus its brevity and the optimal psychometric values, PHQ-9 is a widely used clinical and research tool. PHQ-9 score can range from 0 to 27 since each of the 9 items can be scored from 0 (not at all) to 3 (nearly every day). Although 10 is often recommended as the cut-off score, the optimal cut-off score may differ depending on the setting. As a severity measure, the PHQ-9 scores of 5, 10, 15, and 20 represent valid and easy-to-remember thresholds demarcating the lower limits of mild, moderate, moderately severe, and severe depression. In particular, scores less than 10 seldom occur in individuals with major depression while scores of 15 or greater usually signify the presence of major depression. We adopted the Italian version developed by Picardi and colleagues [32].

3. A specialist psychiatric feedback of second level evaluation results is sent to the occupational physician who, if tests are indicative of an impairment in psychological functioning, proposes to the worker a specialist consultation in person. That third-level evaluation is comprised by the specialist consultation within one week from the second level evaluation, and is followed, according to each single case, by an eventual psychiatric follow-up or psychotherapy.

4. Prospective re-evaluation. To individuate late signs of psychological distress, all subjects in complete psychological wellbeing at first-level evaluation repeat tests after 12 months. All subjects who completed second level questionnaire (indicating sub-optimal psychological wellbeing) at the beginning of the study are re-evaluated after 6 months to assess individual, time-dependent variations over time and to monitor the effectiveness of the ongoing psychological and psychiatric support.

### Data management, statistical analyses, and study endpoints

Data are collected through an automatic database generated by REDCap platform [33], which is subsequently analyzed by STATA software (Stata corp, version 14, Austin, US). An independent coded dataset accessible only to the PI guarantees data protection linking individual information (i.e. name and surname) with an alphanumeric code.

Our primary endpoint is to calculate the percentage of subjects with a total score higher than the cutoff for each of the three first-level tests (GHQ-12, IES-R, GAD-7). Secondary endpoints include the percentage of subjects with specific symptoms at the second level questionnaire (including subscales) and the observed intra-subjects variation after the follow-up re-evaluation (after 6 or 12 months).

Statistical analysis is aimed to individuate risk factors for sub optimal psychological wellbeing and/or impaired psychological function. Potential risk factors include occupational exposure to COVID-19 patients (dividing workers in term of departments, intensity of care of COVID-19 patients, length of job in COVID-19 departments), age, gender, job title and job seniority, non-occupational exposure to COVID-19 (previous positivity to COVID-19 swab, previous COVID-19 cases within workers’ family), individual concerns or believes regarding COVID-19 (including ethical dilemma).

In univariate analyses, comparisons across different groups/levels of COVID-19 exposure are performed through Chi square test to assess differences in the percentage of subjects with a total score higher than the cutoff for each of the three first-level tests, or by parametric (independent samples t-test and one way ANOVA) and non-parametric (e.g. Wilcoxon test) tests for each psychometric scale treated as continuous variables.

In multivariate analysis each potential risk factor is included in multiple logistic regression models to explore the relative contributions (in term of Odds Ratios-OR) of the various risk factors to the dependent variables including potential covariates and confounders.

A *p* < 0.05 will be considered statistically significant. OR are calculated with their relative 95% confidence intervals.

Finally, we will compute regression analysis for repeated measures to evaluate the longitudinal path of primary endpoints over time. Treatment efficacy of psychiatric and psychological support will be assessed through a comparison of the time trend variations of mental health indicators between subjects who underwent treatment compared to the untreated subjects.

## Results

As of December 31, 2020, 550 subjects took part in the study; preliminary results and analysis related to this sample size are shown below.

Participation rate in the first six months resulted about 90%. Table 1 reports sociodemographic variables and exposure experience of COVID-19 of the total sample. As a representative sample of the Italian health care workers general population, participants were predominantly female (65%) with a mean age of 45 years (SD = 11, min = 23, max = 69), the average job seniority (15 years; SD = 11) coincides with that in the current hospital, indicating few previous experiences in different hospital. Nurses (40%) resulted the largest job category followed by physicians (30%). Almost one-third of all participants (*N* = 158, 28.9%) reported to suffer from one disease, 4.9% (*N* = 27) from two, and only 0.4% (*N* = 2) had three or more chronic diseases. Most frequent diseases were arterial hypertension and cardiac (11.3%) and endocrine and metabolic disorders (7.6%). Two hundred and five (37%) subjects reported to regularly take medication, mainly anxiolytics, sedatives, antidepressants or sleeping pills (8,5%), antihypertensive (8%) and pain relievers, analgesics, anti-inflammatories (5%); among these, 56 (10% of the total sample) declared to have started their medication after the pandemic onset: specifically, subjects started assuming mostly anxiolytics/sedatives/antidepressants/sleeping pills (66% of the overall declared use) and vitamins (81% of the overall declared use) after March 2020. At enrolment, 325 (59%) participants had experience of working in a COVID-19 area: 41% was still working with COVID-19 patients and 23% had previously worked in a COVID-19 department (for example, during the first wave of the pandemic).

**Table 1:** Sociodemographic characteristics and exposure experience of COVID-19

Total sample *N* = 550	N	%
Female	353	46
Male	197	36
**Age group**		
20–30	73	13
31–40	149	27
41–50	135	25
51–60	148	27
> 60	45	8
**Occupational role**		
Physician	164	29
Nursing staff	222	40
Health assistant	33	6
Administrative staff	60	11
Others^a^	71	13
**Chronic disease**	189	34
**Positive to nasopharyngeal swab**	60	11
**Experience of quarantine**	90	16
**COVID-19 area working experience**		
*Never*	225	41
*Previous*	125	23
*Current*	200	36
**Colleague infected**	455	83
**Family member infected**	75	14
**Family member hospitalized (due to COVID-19)**	18	3
**Family member deceased (due to COVID-19)**	10	2
**Previous professional experiences with infectious diseases**	209	38
**Specific training on Personal Protective Equipment (PPE)**	395	72
	**Mean**	**SD**
**Seniority of current occupational role (years)**	15	11
**Working years in the current Hospital (years)**	15	11
**Overall working years in the healthcare system (years)**	18	11

^a^ “others” job categories include healthcare technicians, *N* = 55, 10%; auxiliary technicians, *N* = 15, 3%; psychologists, *N* = 1, 0.2%

Table 2 shows health beliefs and COVID-19 related concerns of the total sample. Most subjects reported to experience or having experienced worries about the possibility to infect their family members (78%) and changes in their family habits due to the pandemic (70%). About half of the sample (43%) had feelings of fear for their own safety, and a fifth reported experiences of moral injury (20%) and thoughts about leaving the job (19%).

**Table 2:** Health beliefs and COVID-19 related concerns in the whole sample

Total sample *N* = 550	N	%
**Worries of infecting family**		
Not at all	33	6
Little	85	16
Enough	194	35
Very	238	43
**Having felt discriminated as HCW**		
Not at all	282	51
Little	159	29
Enough	73	13
Very	36	7
**Having felt physically avoided as HCW**		
Never	283	51
Occasionally	197	36
Often	66	12
Always	4	1
**Changes in family’s habits**		
Not at all	49	9
Little	123	22
Enough	177	32
Very	201	37
**Having thought about changing jobs**		
Not at all	361	66
Little	84	15
Enough	68	12
Very	37	7
**Fear for self-safety**		
Not at all	92	17
Little	218	40
Enough	171	31
Very	69	12
**Moral injury (for workers with COVID-19 area working experience *N* = 324)**		
Not at all	134	41
Little	89	28
Enough	76	23
Very	25	8

Table 3 shows results of first and second-level questionnaire scales. At the end of the first level questionnaire, 39% of participants scored above the GHQ cut-off point, suggesting a general psychological distress, and about one-fifth of the subjects had post-traumatic stress symptoms and general anxiety manifestation.

**Table 3:** Mean, standard deviation, range and cut-off score of questionnaires scales

	**Mean**	**Sd**	**Range**	**Cutoff**	**% > Cutoff**
GHQ-12 (*N* = 550)	3.35	3.32	0 − 12	4	39%
IES-R (*N* = 550)	20.56	17.40	0 − 88	33	22%
GAD-7 (*N* = 550)	5.84	5.26	0 − 21	10	21%
PSC4 (*N* = 550)	3.02	0.89	1—5	-	-
			0 − 27	11	34%^a^
DES (*N* = 192)	11.80	12.39	0 − 100	20	20%^a^
SCL-90 (*N* = 192)	0.78	0.60	0 − 4	1	27%^a^

^a^percentage for the second levels questionnaires are calculated on the total of subject who answered the second level, *N* = 192 (i.e. not on the total sample of 550)

Among 192 participants (35% of the total sample) who resulted positive to psychological discomfort and subsequently filled the second level questionnaire, prevalent symptoms were depressive (34% positive at PHQ-9 scale, corresponding to 12% of all enrolled workers), while 20% (equal to 7% of total the sample) resulted above the cut off in the DES scale and 27% (9% of the total) in the SCL-90 questionnaire (at least one subscale).

Table 4 collects results of the first level univariate analysis. According to Chi-square test, the percentage of subjects scoring above the cutoff of the first level scales significantly differs by gender, age, occupational role, COVID-19 exposure at work and in their own family, presence of chronic diseases. Similar results are found in mean differences in each scale scoring, tested through t-test and one-way ANOVA. No statistically significant differences are found considering experience of quarantine or self-infection.

**Table 4:** First level scales across subgroups: means, standard deviations and frequencies of scorings above the cutoff. T-test and ANOVA for continuous variables, Chi-square test for categorical variables

	**GHQ-12**		**IES-R**		**GAD-7**	
	**Mean (sd)**	**N(%) > cutoff**	**Mean (sd)**	**N(%) > cutoff**	**Mean (sd)**	**N(%) > cutoff**
Male	2.90 (3.05)	69 (35)	16.7 (15.6)	32 (16)	4.55 (4.40)	28 (14)
Female	3.67 (3.45)	148 (42)	23.0 (18.1)	88 (25)	6.66 (5.61)	88 (25)
	*		***	*	***	**
**Age group**						
20–30	4.39 (3.79)	38 (53)	23.2 (19.6)	22 (31)	6.77 (5.20)	18 (25)
31–40	3.76 (3.35)	68 (45)	21.7 (16.3)	36 (24)	6.42 (5.17)	35 (23)
41–50	3.17 (3.29)	44 (32)	19.9 (18.4)	27 (20)	5.69 (5.51)	28 (20)
51–60	3.29 (3.28)	57 (38)	22.3 (17.8)	34 (23)	6.02 (5.62)	33 (22)
> 60	1.55 (1.79)	8 (17)	10.4 (8.53)	1 (2)	2.84 (2.65)	1 (2)
	***	**	***	**	***	*
**Occupational role**						
Administrative staff	2.19 (2.63)	15 (24)	16.2 (12.1)	5 (8)	4.35 (4.20)	6 (9)
Health assistant	2.88 (3.43)	10 (30)	24.5 (17.3)	8 (24)	6.66 (5.65)	10 (30)
Nursing staff	4.19 (3.70)	106 (48)	25.1 (20.0)	70 (31)	6.73 (5.77)	58 (26)
Physician	3.04 (2.94)	62 (38)	16.6 (14.4)	23 (14)	5.33 (4.81)	29 (17)
Others	3.00 (2.98)	24 (34)	18.9 (16.2)	14 (19)	5.55 (5.16)	13 (18)
	***	**	***	***	**	*
**Chronic disease**						
Yes	3.57 (3.50)	78 (41)	22.9 (19.2)	51 (27)	6.39 (5.83)	50 (26)
No	3.29 (3.25)	139 (38)	19.6 (16.4)	69 (19)	5.64 (4.99)	66 (18)
			*	*		*
**Positive nasopharyngeal swab**						
Yes	3.37 (3.60)	23 (39)	20.0 (16.3)	15 (25)	5.91 (4.76)	9 (15)
No	3.39 (3.30)	194 (39)	20.8 (17.6)	105 (21)	5.90 (5.37)	107 (21)
**Experience of quarantine**						
Yes	3.67 (3.53)	41(46)	22.2 (17.7)	25 (28)	6.28 (4.63)	19 (21)
No	3.34 (3.29)	176 (38)	20.4 (17.4)	95 (20)	5.82 (5.42)	97 (21)
**COVID-19 area working experience**						
Never	2.53 (2.77)	63 (28)	16.1 (14.1)	28 (12)	4.72 (4.77)	31 (13)
Yes^†^						
*currently*	4.20 (3.65)	100 (50)	25.1 (19.3)	62 (31)	7.20 (5.51)	58 (29)
*previously*	3.66 (3.40)	54 (43)	21.8 (17.9)	30 (24)	5.94 (5.40)	27 (21)
	***	***	***	***	***	***
> *120 days*	4.17 (3.62)	78 (49)	25.1 (19.1)	50 (32)	7.14 (5.64)	47 (30)
< *120 days*	3.88 (3.52)	71 (45)	22.4 (18.5)	38 (24)	6.35 (5.44)	37 (23)
	***	***	***	***	***	***
*high-intensity area*	4.18 (3.60)	132 (50)	25.2 (19.4)	81 (31)	6.96 (5.60)	72 (27)
*low-intensity area*	3.20 (3.26)	22 (35)	18.1 (14.9)	11 (17)	5.67 (4.97)	13 (21)
	***	***	***	***	***	***
**Family member positive to COVID-19**						
Yes	4.44 (3.36)	44 (58)	23.9 (16.9)	24 (32)	7.30 (5.10)	23 (30)
No	3.23 (3.30)	173 (36)	20.2 (17.5)	96 (20)	5.68 (5.30)	93 (19)
	**	***		*	*	*

^*^p < 0.05; **p < 0.005; *** *p* < 0.001

^†^*p* values refer to comparisons between subjects with working experiences in COVID-19 area ( current/previous, number of days, intensity area) and subjects with no experience in COVID-19 area

Table 5 shows univariate analysis for the second level scales, which were completed by 192 subjects. Similarly to firs-level screening, gender and occupational role result as statistically significant factors associated to psychological distress: means and percentage of scoring above the cutoff are higher for females, nurses and health assistants although the latter are few cases. Contrary to first-level outcomes, age, COVID-19 personal and working exposure lose their association with psychological scales.

**Table 5:** Second level scales across subgroups (*N* = 192): means, standard deviations and frequencies of scorings above the cutoff. T-test and ANOVA for continuous variables, Chi-square test for categorical variables

		**PHQ-9**		**DES**		**SCL-90**	
	**N (%)**	**Mean (sd)**	**N(%) > cutoff**	**Mean (sd)**	**N(%) > cutoff**	**Mean (sd)**	**N(%) > cutoff**
Male	57 (30)	8.43 (4.79)	12 (21)	9.19 (8.74)	9 (15)	0.61 (0.42)	9 (16)
Female	135 (70)	9.61 (5.76)	51 (37)	12.8 (13.3)	29 (21)	0.83 (0.65)	41 (30)
			*	*		**	*
**Age group**							
20–30	34 (18)	10.2 (5.79)	11 (32)	12.7 (10.8)	7 (20)	0.87 (0.65)	12 (35)
31–40	59 (31)	8.64 (5.31)	14 (23)	13.6 (12.5)	17 (28)	0.73 (0.57)	17 (29)
41–50	44 (23)	9.34 (6.19)	17 (38)	9.75 (10.4)	6 (13)	0.74 (0.59)	10 (23)
51–60	48 (25)	9.85 (5.01)	21 (43)	11.6 ( 14.8)	8 (16)	0.82 (0.63)	11 (23)
> 60	5 (3)	4.40 (1.51)	0	3.89 (2.48)	0	0.37 (0.26)	0
**Occupational role**							
Administrative staff	10 (5)	8.10 (4.28)	4 (40)	10.5 (11.1)	2 (20)	0.75 (0.63)	4 (40)
Health assistant	9 (5)	13.0 (5.17)	7 (77)	23.8 (23.9)	5 (55)	1.47 (1.02)	5 (55)
Nursing staff	101 (53)	10.6 (5.70)	36 (35)	14.0 (12.5)	27 (26)	0.86 (0.60)	33 (33)
Physician	49 (25)	7.18 (4.70)	9 (18)	6.87 (6.81)	2 (4)	0.53 (0.33)	4 (8)
Others	23 (12)	6.95 (4.28)	7 (30)	8.19 (8.73)	2 (8)	0.59 (0.54)	4 (17)
		***	**	***	***	***	**
**Chronic disease**							
Yes	71 (37)	10.4 (5.50)	33 (46)	13.2 (13.1)	16 (22)	0.87 (0.63)	22 (31)
No	121 (63)	8.57 (5.41)	30 (24)	10.9 (11.6)	22 (18)	0.71 (0.57)	28 (23)
		*	**				
**Positive nasopharyngeal swab**							
Yes	23 (12)	9.39 (4.44)	7 (30)	10.8 (11.4)	3 (13)	0.67 (0.37)	3 (13)
No	169 (88)	9.24 (5.64)	56 (33)	11.9 (12.3)	35 (20)	0.78 (0.62)	47 (28)
**Experience of quarantine**							
Yes	34 (18)	9.38 (4.52)	9 (26)	10.9 (9.73)	3 (8)	0.76 (0.43)	8 (23)
No	158 (82)	9.24 (5.70)	54 (34)	11.9 (12.7)	35 (22)	0.77 (0.63)	42 (26)
**COVID-19 area working experience**							
Never	53 (28)	8.39 (5.18)	19 (35)	10.5 (11.0)	7 (13)	0.73 (0.62)	12 (22)
Yes^†^							
*currently*	96 (50)	9.69 (5.74)	31 (32)	12.7 (13.3)	20 (21)	0.83 (0.59)	30 (31)
*previously*	43 (22)	9.37 (5.33)	13 (30)	11.1 (11.0)	11 (26)	0.68 (0.57)	8 (19)
> *120 days*	79 (41)	9.77 (5.52)	24 (31)	12.2 (13.3)	15 (19)	0.84 (0.60)	24 (31)
< *120 days*	60 (31)	9.91 (5.66)	19 (33)	13.1 (12.1)	16 (28)	0.76 (0.57)	14 (25)
*high-intensity area*	121 (63)	9.76 (5.60)	38 (31)	12.3 (13.0)	25 (20)	0.77 (0.57)	32 (26)
*low-intensity area*	18 (9)	8.50 (5.63)	6 (33)	11.7 (9.85)	6 (33)	0.84 (0.70)	6 (33)
**Positive family member**							
Yes	38 (20)	9.13 (4.58)	10 (26)	10.2 (8.16)	4 (10)	0.72 (0.48)	7 (18)
No	154 (80)	9.29 (5.72)	53 (34)	12.1 (13.0)	34 (22)	0.78 (0.62)	43 (28)

^*^p < 0.05; **p < 0.005; *** *p* < 0.001

^†^*p* values refer to comparisons between subjects with working experiences in COVID-19 area ( current/previous, number of days, intensity area) and subjects with no experience in COVID-19 area

**Fig. 2:**
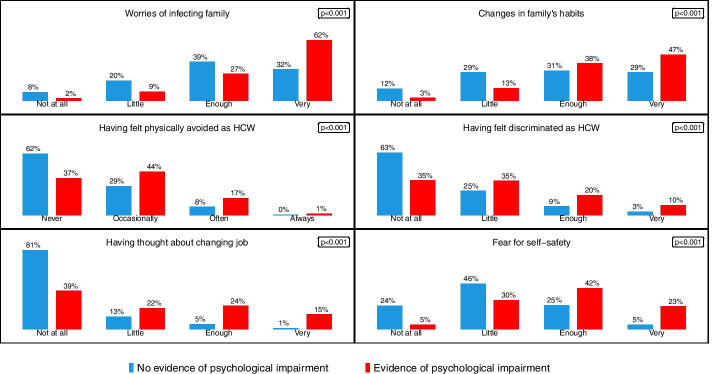
Health beliefs and COVID-19 related concerns: percentage of each answer according to first level screening results (subjects with evidence of psychological impairment versus subjects with no evidence of psychological impairment)

Figure 2 illustrates differences in distribution of health beliefs and COVID-19 concerns for each answers according to first level screening result; worries, discomfort and fear were expressed more frequently by subjects who scored above the cut-off in at least one scale compared to colleagues with no evidence of psychological impairment.

We are currently extending sample size with an ongoing data collection to allow forthcoming risk factor analysis (i.e. multivariate logistic regression).

## Discussion

This paper describes the methodology adopted to assess the psychological health during COVID-19 pandemic, in the context of occupational health surveillance of healthcare workers of a large University Hospital in Milan, Italy. We decided to systematically assess the psychological health of all employees, in order to obtain the widest range of exposure to the pandemic and to infected patients, and also to properly assess the impact of non-occupational risk factors in determining anxiety, depressed mood or post-traumatic symptoms.

The above mentioned setting of intervention, with the possibility to offer specialist treatment when needed, seemed to having maximized the participation rate of the workers and, on the same time, it made us able to properly evaluate and take care of a narrow span of psychological symptoms and/or disorders.

In the current methodological paper, we presented first results based on data collected from August 01, to December 31, 2020, when SARS-CoV-2 vaccine started being administered to healthcare workers of our Hospital. This span of time has been chosen consciously, in order to gather all the assessments before the availability of the vaccine and then to compare data previous and after the completion of vaccinations, reached after only two months.

Univariate analysis suggests that potential risk factors for psychological impairment are gender, age, occupational role, chronic conditions as well as working and private life exposure to COVID-19: women, nurses, young workers and subjects directly involved in COVID-19 areas or with an infected family member expressed significantly less psychological wellbeing compared to colleagues. Among all subjects directly engaged with COVID-19 patients, those who had concomitant, longer than 120 days, and high-intensity unit experience in COVID-19 area at time of enrollment showed higher scores on all three first-level scales compared to colleagues with dissimilar COVID-19 working experience.

Differently from colleagues who did not show psychological impairment, participants referred to the second level evaluations also reported more concerns about infecting their family and self-safety, felt more discriminated as HCWs, expressed intention to leave their job. Forthcoming analysis are needed to better explore these associations.

Considering second-level evaluation, personal and working COVID-19 exposures are no longer significantly associated to psycho-diagnostic scales. Potential risks factors for psychological distress may be pre-existing characteristics as gender, occupational role and previous medical condition. Multivariate analysis will increase our knowledge on the relative contributions of each risk factors to HCWs mental health.

As all questionnaire-based studies, our data are based on a self-reported methodology which makes data validity consequent to participants’ motivation and reliability. In order to minimize this limitation, we used self-reported measures integrated with medical evaluations (i.e. at first-level the occupational physician completes data collection and computes scales values, and at second- and third-level assessments a psychiatrist or a psychologist examines and evaluates symptoms).

Another limitation of the study is the lack of previous data about the psychological wellbeing in the population of health care workers before the pandemic onset. Thus, our finding should be carefully managed and not all the symptoms should be interpreted as caused by the exposure to the pandemic effects and consequences. Even the lack of previous systematic psychological research among Italian healthcare population during a pandemic implicates a degree of uncertainty in predicting psychological impairment in our sample size, and also in comparing results at a national level.

## Conclusion

Literature is showing the psychological burden of COVID-19 pandemic on hospital workers is substantial, however most research pertain to cross-sectional studies aimed at detecting reactions occurred in the first months of the health emergency. Our methodology emphasizes preventive and rehabilitation measures by providing an ongoing evaluation and follow-up interventions to analyze and support psychological wellbeing of all employees, directly or indirectly exposed to pandemic consequences; moreover, this methodology constitutes a unique condition to detect individual, occupational, and non-occupational risk factors for psychological impairment in situations of high stress and/or disasters.

## Data Availability

The dataset generated and analysed during the current study are not publicly available due to restrictions related to our internal review board and to our hospital policy in relations to public health workers but are available from the Principal Investigator (Matteo Bonzini) on reasonable request.

## Abbreviations

ANOVA: Analysis of Variance
COVID-19: Coronavirus Disease 2019
DES: Dissociative Experiences Scale
DSM-IV: Diagnostic and Statistical Manual of Mental Disorders, Fourth Edition
GAD-7: 7 Items Generalized Anxiety Disorder Scale
GHQ: General Health Questionnaire
IES-R: Impact of Event Scale- Revised
PHQ-9: Patient Health Questionnaire
PI: Principal Investigator
PSC: Psychosocial Safety Climate
PTSD: Post-traumatic Stress Disorder
SARS-CoV-2: Severe Acute Respiratory Syndrome Coronavirus 2
SCL: Symptoms Check List
